# Advancing the Design of Observational Studies in Drug‐Coated Balloon Angioplasty: Bridging Practice With Evidence‐Based Standards

**DOI:** 10.1002/clc.70238

**Published:** 2025-12-15

**Authors:** Kunal Mahajan, Jaibharat Sharma, Surender Himral, Vivek Rana, Shekhar Vohra

**Affiliations:** ^1^ Himachal Heart Institute Mandi Himachal Pradesh India; ^2^ Innocent Heart Hospital Jalandhar Punjab India; ^3^ Mount Sinai Morningside Icahn School of Medicine at Mount Sinai New York City New York USA

Dear Editor,

We read with great interest the observational study by Meunier et al. evaluating a stent‐less strategy using drug‐coated balloons (DCB) in coronary artery disease (CAD) [1]. The present single‐center observational study evaluating the long‐term effectiveness of a stent‐less strategy with DCB angioplasty in CAD undoubtedly adds to the literature regarding contemporary percutaneous coronary intervention (PCI) [1]. Nevertheless, methodological limitations remain that could impact the reliability and generalizability of the findings, which were not fully addressed in the manuscript. Most critically, the absence of randomization and reliance on operator discretion for allocating stent‐less versus bailout drug‐eluting stent (DES) strategies raises concerns regarding selection bias and confounding. Randomized controlled trials (RCTs) such as BASKET‐SMALL 2 and PICCOLETO II have demonstrated the importance of random allocation in minimizing selection bias and ensuring comparability across intervention arms, thereby providing more robust estimates of relative effectiveness between DCB and DES strategies [2, 3]. Employing a multicenter randomized design, as recommended in recent consensus statements, would also enhance external validity and facilitate application to broader clinical practice [4, 5]. Another key limitation relates to the lack of core laboratory adjudication and systematic use of intravascular imaging for lesion assessment and procedural outcome validation. RCTs and multicenter DCB registries increasingly mandate independent angiographic review and intravascular imaging such as OCT or IVUS to rigorously evaluate lesion morphology, residual stenosis, and dissection severity [4]. Studies have shown that core lab adjudication substantially reduces inter‐observer variability and measurement bias, thereby improving endpoint ascertainment and clinical trial integrity [5, 6]. Furthermore, the manuscript provides insufficient detail regarding standardization and stratification of concomitant antiplatelet therapy, which may have influenced outcome measurements such as major adverse cardiac events and bleeding rates. The heterogeneity in antiplatelet regimens was noted but not adjusted for in the primary analyses. Recent RCTs including TWILIGHT and TICO have demonstrated that clearly defined, protocolized antiplatelet regimens and stratified reporting are essential for interpreting ischemic and bleeding risk profiles in PCI populations [7, 8]. To address these limitations, future studies should prioritize multicenter RCT designs with independent core laboratory angiographic adjudication and routine use of contemporary intravascular imaging. Endpoints must be standardized according to consensus definitions, with adjustment for confounders including antiplatelet therapy regimens, in line with recommendations from leading international PCI trials and expert statements [2, 3, 4, 5, 6]. Only by adopting these rigorous methodological measures can the relative merits of stent‐less PCI strategies be definitively established for broad clinical practice.

## Author Contributions

All authors contributed to the writing of the correspondence and have approved the final version.

## Ethics Statement

The authors have nothing to report.

## Conflicts of Interest

The authors declare no conflicts of interest.

## Data Availability

Data sharing is not applicable to this article as no data sets were generated or analyzed during the current study.
